# Deflection of Cross-Ply Composite Laminates Induced by Piezoelectric Actuators

**DOI:** 10.3390/s100100719

**Published:** 2010-01-20

**Authors:** Shiuh-Chuan Her, Chi-Sheng Lin

**Affiliations:** Department of Mechanical Engineering, Yuan-Ze University, Chung-Li, Taiwan; E-Mail: s917224@mail.yzu.edu.tw

**Keywords:** piezoelectric actuator, bending moment, plate theory, flexural displacement

## Abstract

The coupling effects between the mechanical and electric properties of piezoelectric materials have drawn significant attention for their potential applications as sensors and actuators. In this investigation, two piezoelectric actuators are symmetrically surface bonded on a cross-ply composite laminate. Electric voltages with the same amplitude and opposite sign are applied to the two symmetric piezoelectric actuators, resulting in the bending effect on the laminated plate. The bending moment is derived by using the classical laminate theory and piezoelectricity. The analytical solution of the flexural displacement of the simply supported composite plate subjected to the bending moment is solved by using the plate theory. The analytical solution is compared with the finite element solution to show the validation of present approach. The effects of the size and location of the piezoelectric actuators on the response of the composite laminate are presented through a parametric study. A simple model incorporating the classical laminate theory and plate theory is presented to predict the deformed shape of the simply supported laminate plate.

## 1. Introduction

Piezoelectric materials with the advantages of quick response, low power consumption and high linearity have drawn much attention in the past decade. Piezoelectric devices are of great interest in structural engineering with applications to shape control, vibration suppression and noise reduction [1]. Smart structures integrated with sensors and actuators have the capability to respond to a changing environment and control the structural movement. Piezoceramics are the most common material used in smart structures and can be surface bonded to existing structures to form an online monitoring system, or embedded in composite structures without significantly changing the structural stiffness system. Bailey and Hubbard [2] developed first adaptive structure using polyvinylidene fluoride (PVDF) film as actuators to control the structural vibration of a cantilever beam. Crawley and de Luis [3] studied a beam with surface bonded and embedded piezoelectric actuators to investigate the load transfer between the actuator and host beam. Huang and Sun [4] studied the load transfer and wave propagation of an anisotropic elastic medium induced by the surface bonded piezoelectric actuator. Dimitriadis *et al.* [5] used two dimensional patches of piezoelectric material bonded to the surface of a simply supported plate as vibration actuators to excite the selected modes. Shape control is one of the major applications for piezoelectric materials. Koconis *et al.* [6] controlled the shape of composite plates and shells with piezoelectric actuators. Luo and Tong [7] developed a finite element model to simulate twisting and bending shape control using the orthotropic piezoelectric actuators. Lin and Nien [8] used piezoelectric actuators to control the deflection and shape of the composite laminates. Other works related to the shape control of composites with piezoelectric actuators were presented by Bulter *et al.* [9,10]. The finite element method, a widely accepted and powerful tool for analyzing complex structures, is capable of dealing with the piezoelectric smart structures. Numerous studies have been completed on analyzing piezoelectric structures to account for the piezoelectric effect [11–13]. Actuators used in active control of smart structures have to be located appropriately to ensure maximum control and measurement effectiveness. The methods for optimal placement of sensors and actuators were investigated by many researchers [14–16].

The present work investigated the load transfer between surface bonded piezoelectric actuators and the host structure. The proposed method is an extension of the one dimensional beam embedded with PZT derived by Crawley and de Luis [3] and two dimensional plate surface bonded with PZT derived by Dimitriadis *et al.* [5]. The model consists of two piezoelectric actuators symmetrically surface bonded on a cross-ply composite laminate subjected to electrical voltage. An analytical expression of the bending moment induced by the piezoelectric actuators was derived by incorporating the composite laminate theory and piezoelectric effect. The bending moment was then applied to the composite laminate with simply supported boundary conditions. A closed form solution to the deflection of the simply supported composite laminate was obtained by using the plate theory. The analytical solution was validated with the finite element results. Finite element method has been widely used in structural analysis. It is reasonable to verify present approach with finite element method. The effects of the size and location of the piezoelectric actuators on the response of the composite laminate are presented through a parametric study. The objective of this investigation is to develop an analytical expression of the response of a thin plate excited by the bonded piezoelectric actuators. A simple model incorporating the classical laminate theory and plate theory is proposed to predict the deformation of the composite laminate plate. The feasibility of controlling the deflected shape of the plate is illustrated by placing the actuators at various locations.

## 2. Bending Moment

Consider two piezoelectric actuators symmetrically surface bonded on a cross-ply composite laminate. The polarized direction is along the z-axis. For an unconstrained thin piezoelectric actuator, equal strains in both *x* and *y* directions will be induced when activated by a voltage along the poling direction. The magnitude of the strain can be expressed in terms of the piezoelectric constant *d*_31_, applied voltage *V* and actuator thickness *t_pe_*, as follows:

(1) $${\left(\varepsilon_{x}\right)}_{\mathrm{pe}}={\left(\varepsilon_{y}\right)}_{\mathrm{pe}}=\varepsilon_{\mathrm{pe}}=\frac{d_{31}}{t_{\mathrm{pe}}}V$$

where subscripts *pe* and *p* represent the quantities associated with piezoelectric actuator and host plate, respectively, throughout this paper. When an electrical field is applied in the direction normal to the actuator surface, surface strains are generated Equation (1). Due to the coupling of the actuator to the structure, forces and moments are induced in the bonded area of the structure. Since this work focus on the deformation of the plate induced by the bending moment, only constant d31 is considered in this model.

**Figure 1.**
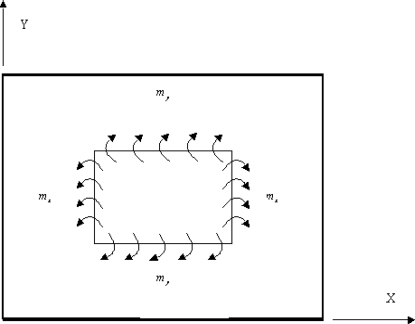
Bending moment acting on the composite laminate induced by the lead zirconate titanate PZT actuators.

The two actuators are activated by applying a voltage of equal magnitude and opposite sign to the opposing actuators. The opposite directions of the surface tractions at the interfaces between the actuator and plate cause the uniform bending moments along the actuator boundaries as shown in Figure 1.

**Figure 2.**
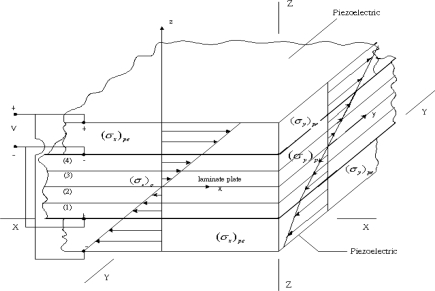
Strain distribution across the thickness of the composite laminate.

The symmetry of the actuators with respect to the midplane (*z* = 0) results in no net extension or contraction in the midplane of the plate. In the following derivation, the piezoelectric actuators are assumed to be perfectly bonded to the composite laminate and in the state of plane stress. This implies the strain continuity across the interfaces as shown in Figure 2.

The strains across the thickness of the composite laminate due to the bending moment can be expressed as follows:

(2) $$\varepsilon_{x}={Z\kappa }_{x};\;\;\;\;\;\;\;\;\varepsilon_{y}={Z\kappa }_{y};\;\;\;\;\;\;\;\;\varepsilon_{\mathrm{xy}}={Z\kappa }_{\mathrm{xy}}$$

where Z is the position along the thickness measured from the midplane of the laminate, κ*_x_*, κ*_y_* and κ*_xy_* are the curvatures of the composites laminate.

The bending stresses in the k-th layer of the composite laminate are:

(3) $${\left\{\begin{array}{c} \sigma_{x}\\ \sigma_{y}\\ \tau_{\mathrm{xy}} \end{array}\right\}}^{k}=Z{\left[\begin{array}{ccc} {\bar{Q}}_{11} & {\bar{Q}}_{12} & {\bar{Q}}_{16}\\ {\bar{Q}}_{12} & {\bar{Q}}_{22} & {\bar{Q}}_{26}\\ {\bar{Q}}_{16} & {\bar{Q}}_{26} & {\bar{Q}}_{66} \end{array}\right]}^{k}\left\{\begin{array}{c} \kappa_{x}\\ \kappa_{y}\\ \kappa_{\mathrm{xy}} \end{array}\right\}$$

$$\begin{array}{l} {\bar{Q}}_{11}=Q_{11}\;{\text{cos}}^{4}\;\theta +2\left(Q_{12}+{2Q}_{66}\right){\text{sin}}^{2}\;\theta \;{\text{cos}}^{2}\;\theta +Q_{22}\;{\text{sin}}^{4}\;\theta \\ {\bar{Q}}_{12}=\left(Q_{11}+Q_{22}-{4Q}_{66}\right){\text{sin}}^{2}\;\theta \;{\text{cos}}^{2}\;\theta +Q_{12}\;\left({\text{sin}}^{4}\;\theta \;{\text{cos}}^{4}\;\theta \right)\\ {\bar{Q}}_{22}=Q_{11}\;{\text{sin}}^{4}\;\theta +2\left(Q_{12}+{2Q}_{66}\right){\text{sin}}^{2}\;\theta \;{\text{cos}}^{2}\;\theta +Q_{22}\;{\text{cos}}^{4}\;\theta \\ {\bar{Q}}_{16}=\left(Q_{11}-Q_{12}-{2Q}_{66}\right)\text{sin}\;\theta \;{\text{cos}}^{3}\;\theta +\left(Q_{12}-Q_{22}+{2Q}_{66}\right){\text{sin}}^{3}\;\theta \;\text{cos}\;\theta \\ {\bar{Q}}_{26}=\left(Q_{11}-Q_{12}-{2Q}_{66}\right){\text{sin}}^{3}\;\theta \;\text{cos}\;\theta +\left(Q_{12}-Q_{22}+{2Q}_{66}\right)\text{sin}\;\theta \;{\text{cos}}^{3}\;\theta \\ {\bar{Q}}_{66}=\left(Q_{11}+Q_{22}-{2Q}_{12}-2Q_{66}\right){\text{sin}}^{2}\;\theta \;{\text{cos}}^{2}\;\theta +Q_{66}\;\left({\text{sin}}^{4}\;\theta +{\text{cos}}^{4}\;\theta \right)\\ Q_{11}=\frac{E_{1}}{1-\nu_{12}\nu_{21}}\\ Q_{12}=\frac{\nu_{12}E_{2}}{1-\nu_{12}\nu_{21}}\\ Q_{22}=\frac{E_{2}}{1-\nu_{12}\nu_{21}}\\ Q_{66}=G_{12} \end{array}$$

where *E*_1_ and *E*_2_ are the Young’s moduli along the fiber direction and normal to the fiber direction, respectively. *G*_12_ is the shear modulus, *v*_12_ and *v*_21_ are the Poisson’s ratios, *θ* is the fiber orientation measured from the global x-axis.

In the case of cross-ply (*θ* = 0° or 90°), *Q̄*_16_ and *Q̄*_26_ are equal to zero. The bending stresses in the k-th layer can be reduced to:

(4a) $$\sigma_{x}^{\left(k\right)}=Z\left[{\bar{Q}}_{11}^{\left(k\right)}\kappa_{x}+{\bar{Q}}_{12}^{\left(k\right)}\kappa_{y}\right]$$

(4b) $$\sigma_{y}^{\left(k\right)}=Z\left[{\bar{Q}}_{12}^{\left(k\right)}\kappa_{x}+{\bar{Q}}_{22}^{\left(k\right)}\kappa_{y}\right]$$

(4c) $$\sigma_{\mathrm{xy}}^{\left(k\right)}={Z\bar{Q}}_{66}^{\left(k\right)}\kappa_{\mathrm{xy}}$$

The bending stresses in the piezoelectric actuator are:

(5a) $${\left(\sigma_{x}\right)}_{\mathrm{pe}}=\frac{E_{\mathrm{pe}}}{1-\nu_{\mathrm{pe}}^{2}}\left[{Z\kappa }_{x}+\nu_{\mathrm{pe}}{Z\kappa }_{y}-\left(1+\nu_{\mathrm{pe}}\right)\varepsilon_{\mathrm{pe}}\right]$$

(5b) $${\left(\sigma_{y}\right)}_{\mathrm{pe}}=\frac{E_{\mathrm{pe}}}{1-\nu_{\mathrm{pe}}^{2}}\left[{Z\kappa }_{y}+\nu_{\mathrm{pe}}{Z\kappa }_{x}-\left(1+\nu_{\mathrm{pe}}\right)\varepsilon_{\mathrm{pe}}\right]$$

where *E_pe_* and *v_pe_* are the Young’s modulus and Poisson’s ratio of the piezoelectric actuator.

The bending moments per unit length *m_x_* and *m_y_* are defined as the forces *σ_x_dz* and *σ_y_dz* times the moment arm *z*, respectively. The sum of the bending moments with respect to the neutral axis (*z* = 0) across the thicknesses of the composite laminate and piezoelectric actuators are zero, such that:

(6a) $$\int_{-t}^{t}{\left(\sigma_{x}\right)}_{p}\mathrm{ZdZ}+\int_{-h-t}^{-t}{\left(\sigma_{x}\right)}_{\mathrm{pe}}\mathrm{ZdZ}+\int_{t}^{t+h}{\left(\sigma_{x}\right)}_{\mathrm{pe}}\mathrm{ZdZ}=0$$

(6b) $$\int_{-t}^{t}{\left(\sigma_{y}\right)}_{p}\mathrm{ZdZ}+\int_{-h-t}^{-t}{\left(\sigma_{y}\right)}_{\mathrm{pe}}\mathrm{ZdZ}+\int_{t}^{t+h}{\left(\sigma_{y}\right)}_{\mathrm{pe}}\mathrm{ZdZ}=0$$

where *t* is one half of the composite laminate thickness and *h* is the thickness of the piezoelectric actuator.

(7a) $$\left[{\left(D_{11}\right)}_{p}+2{\left(D_{11}\right)}_{\mathrm{pe}}\right]\kappa_{x}+\left[{\left(D_{12}\right)}_{p}+2{\left(D_{12}\right)}_{\mathrm{pe}}\right]\kappa_{y}=\left(1+\nu_{\mathrm{pe}}\right)2{\left(B_{11}\right)}_{\mathrm{pe}}\varepsilon_{\mathrm{pe}}$$

(7b) $$\left[{\left(D_{12}\right)}_{p}+2{\left(D_{12}\right)}_{\mathrm{pe}}\right]\kappa_{x}+\left[{\left(D_{22}\right)}_{p}+2{\left(D_{22}\right)}_{\mathrm{pe}}\right]\kappa_{y}=\left(1+\nu_{\mathrm{pe}}\right)2{\left(B_{11}\right)}_{\mathrm{pe}}\varepsilon_{\mathrm{pe}}$$

$$\begin{array}{l} {\left(D_{11}\right)}_{\mathrm{pe}}={\left(D_{22}\right)}_{\mathrm{pe}}=\frac{1}{3}\frac{E_{\mathrm{pe}}}{1-\nu_{\mathrm{pe}}^{2}}\left({\left(t+h\right)}^{3}-t^{3}\right)\\ {\left(D_{12}\right)}_{\mathrm{pe}}=\frac{1}{3}\frac{\nu_{\mathrm{pe}}E_{\mathrm{pe}}}{1-\nu_{\mathrm{pe}}^{2}}\left({\left(t+h\right)}^{3}-t^{3}\right)\\ {\left(B_{11}\right)}_{\mathrm{pe}}=\frac{1}{2}\frac{E_{\mathrm{pe}}}{1-\nu_{\mathrm{pe}}^{2}}\left({\left(t+h\right)}^{2}-t^{2}\right)\\ {\left(D_{11}\right)}_{p}=\frac{1}{3}\sum_{k=1}^{N}{\bar{Q}}_{11}^{k}\left(Z_{k}^{3}-Z_{k-1}^{3}\right)\\ {\left(D_{22}\right)}_{p}=\frac{1}{3}\sum_{k=1}^{N}{\bar{Q}}_{22}^{k}\left(Z_{k}^{3}-Z_{k-1}^{3}\right)\\ {\left(D_{12}\right)}_{p}=\frac{1}{3}\sum_{k=1}^{N}{\bar{Q}}_{12}^{k}\left(Z_{k}^{3}-Z_{k-1}^{3}\right) \end{array}$$

where *Z_k_* and *Z*_*k*−1_ represent the positions of the top and bottom surfaces of the k-th layer in the composite laminate, respectively.

The curvatures κ*_x_* and κ*_y_* can be solved from Equation (7) as:

(8a) $$\kappa_{x}=A_{1}\varepsilon_{\mathrm{pe}}$$

(8b) $$\kappa_{y}=A_{2}\varepsilon_{\mathrm{pe}}$$

(8c) $$A_{1}=\frac{B_{11\mathrm{pe}}\left(1+\nu_{\mathrm{pe}}\right)}{{\left(D_{11}\right)}_{p}+D_{11\mathrm{pe}}}+\frac{\left(\left({\left(D_{12}\right)}_{p}+D_{12\mathrm{pe}}\right)\left(-B_{11\mathrm{pe}}\left({\left(D_{11}\right)}_{p}+D_{11\mathrm{pe}}\right)\left(1+\nu_{\mathrm{pe}}\right)+B_{11\mathrm{pe}}\left({\left(D_{12}\right)}_{p}+D_{12\mathrm{pe}}\right)\left(1+\nu_{\mathrm{pe}}\right)\right)\right)}{\left(\left({\left(D_{11}\right)}_{p}+D_{11\mathrm{pe}}\right)\left(-{\left({\left(D_{12}\right)}_{p}+D_{12\mathrm{pe}}\right)}^{2}+\left({\left(D_{11}\right)}_{p}+D_{11\mathrm{pe}}\right)\left({\left(D_{22}\right)}_{p}+D_{22\mathrm{pe}}\right)\right)\right)}$$

(8d) $$A_{2}=\frac{-B_{11\mathrm{pe}}\left({\left(D_{11}\right)}_{p}+D_{11\mathrm{pe}}\right)\left(1+\nu_{\mathrm{pe}}\right)+B_{11\mathrm{pe}}\left({\left(D_{12}\right)}_{p}+D_{12\mathrm{pe}}\right)\left(1+\nu_{\mathrm{pe}}\right)}{-{\left({\left(D_{12}\right)}_{p}+D_{12\mathrm{pe}}\right)}^{2}+\left({\left(D_{11}\right)}_{p}+D_{11\mathrm{pe}}\right)\left({\left(D_{22}\right)}_{p}+D_{22\mathrm{pe}}\right)}$$

The bending moments per unit length *m_x_* and *m_y_* acting on the composite laminate as shown in Figure 2 can be calculated:

(9a) $$m_{x}=\sum_{k=1}^{N}\left[\int_{Z_{k-1}}^{Z_{k}}\sigma_{x}^{\left(k\right)}\mathrm{dz}\right]=\sum_{k=1}^{N}\left[\int_{Z_{k-1}}^{Z_{k}}Z\left({\bar{Q}}_{11}^{\left(k\right)}\kappa_{x}+{\bar{Q}}_{12}^{\left(k\right)}\kappa_{y}\right)\mathrm{dz}\right]$$

(9b) $$m_{y}=\sum_{k=1}^{N}\left[\int_{Z_{k-1}}^{Z_{k}}\sigma_{y}^{\left(k\right)}\mathrm{dz}\right]=\sum_{k=1}^{N}\left[\int_{Z_{k-1}}^{Z_{k}}Z\left({\bar{Q}}_{12}^{\left(k\right)}\kappa_{x}+{\bar{Q}}_{22}^{\left(k\right)}\kappa_{y}\right)\mathrm{dz}\right]$$

Substituting Equation (8) into Equation (9), leads to the bending moments:

(10a) $$m_{x}=C_{1}\varepsilon_{\mathrm{pe}}$$

(10b) $$m_{y}=C_{2}\varepsilon_{\mathrm{pe}}$$

$$\begin{array}{l} C_{1}=\left(A_{1}{\left(D_{11}\right)}_{p}+A_{2}{\left(D_{12}\right)}_{p}\right)\\ C_{2}=\left(A_{1}{\left(D_{12}\right)}_{p}+A_{2}{\left(D_{22}\right)}_{p}\right) \end{array}$$

## 3. Deflection of a Simply Supported Composite Plate

**Figure 3.**
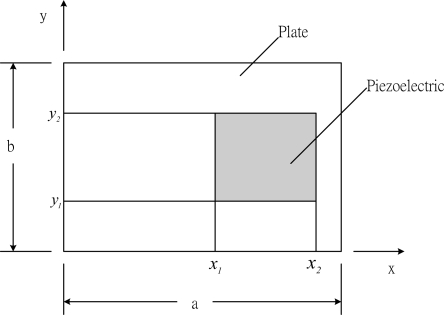
Surface bonded actuator on the composite laminate plate.

A rectangular composite laminate plate with simply supported boundary conditions is considered in this work. The location of the surface bonded actuator is shown in Figure 3. The activated piezoelectric actuators will induce bending moments as derived in Equation (10) to the composite plate and can be expressed in terms of unit step functions as follows:

(11a) $$m_{x}=C_{1}\varepsilon_{\mathrm{pe}}\left[h\left(x-x_{1}\right)-h\left(x-x_{2}\right)\right]\left[h\left(y-y_{1}\right)-h\left(y-y_{2}\right)\right]$$

(11b) $$m_{y}=C_{2}\varepsilon_{\mathrm{pe}}\left[h\left(x-x_{1}\right)-h\left(x-x_{2}\right)\right]\left[h\left(y-y_{1}\right)-h\left(y-y_{2}\right)\right]$$

Using the classical plate theory, the equilibrium equation for the plate can be written in terms of the plate internal moments *M_x_*,*M_y_*,*M_xy_* and the actuators induced moments *m_x_, m_y_* as:

(12) $$\frac{\partial^{2}\left(M_{x}-m_{x}\right)}{{\partial x}^{2}}+2\frac{\partial^{2}M_{\mathrm{xy}}}{\partial x\partial y}+\frac{\partial^{2}\left(M_{y}-m_{y}\right)}{{\partial y}^{2}}=0$$

The internal moments *M_x_*,*M_y_*,*M_xy_* can be expressed in terms of the flexural displacement *w*. Moving the moments *m_x_, m_y_* to the right hand side of Equation (12), yields to the following equilibrium equation of the composite laminate plate:

(13) $$\begin{array}{c} {\left(D_{11}\right)}_{p}\frac{\partial^{4}w}{{\partial x}^{4}}+{2H}_{1}\frac{\partial^{4}w}{{\partial x}^{2}{\partial y}^{2}}+{\left(D_{22}\right)}_{p}\frac{\partial^{4}w}{{\partial y}^{4}}=P\\ H_{1}={\left(D_{12}\right)}_{p}+2{\left(D_{66}\right)}_{p}\\ P=\frac{\partial^{2}m_{x}}{{\partial x}^{2}}+\frac{\partial^{2}m_{y}}{{\partial y}^{2}} \end{array}$$

where (*D*_11_)*_p_*, (*D*_22_)*_p_*, (*D*_66_)*_p_* are the bending stiffness of the composite laminate as defined in Equation (7).

Substituting Equation (11) into Equation (13), leads to the governing differential equation:

(14) $$\begin{array}{c} {\left(D_{11}\right)}_{p}\frac{\partial^{4}w}{{\partial x}^{4}}+{2H}_{1}\frac{\partial^{4}w}{{\partial x}^{2}{\partial y}^{2}}+{\left(D_{22}\right)}_{p}\frac{\partial^{4}w}{{\partial y}^{4}}=C_{1}\varepsilon_{\mathrm{pe}}\left[\delta^{\prime }\left(x-x_{1}\right)-\delta^{\prime }\left(x-x_{2}\right)\right][h(y-y_{1})-h(y-y_{2})\\ \;\;\;\;\;\;\;\;\;+C_{2}\varepsilon_{\mathrm{pe}}\left[h\left(x-x_{1}\right)-h\left(x-x_{2}\right)\right]\left[\delta^{\prime }\left(y-y_{1}\right)-\delta^{\prime }\left(y-y_{2}\right)\right] \end{array}$$

For a simply supported rectangular plate, the flexural displacement *w* can be expressed by the following Fourier series:

(15a) $$w(x,y)=\sum_{m=1}^{\infty }\sum_{n=1}^{\infty }W_{\mathrm{mn}}\;\text{sin}\frac{m\pi x}{a}\;\text{sin}\;\frac{n\pi y}{b}$$

Substituting Equation (15a) into Equation (14), solve for the constant *W_mn_* as follows [5]:

(15b) $$W_{\mathrm{mn}}=\frac{P_{\mathrm{mn}}}{\frac{m^{4}\pi^{4}}{a^{4}}{\left(D_{11}\right)}_{p}+\frac{m^{2}\pi^{2}}{a^{2}}\frac{n^{2}\pi^{2}}{b^{2}}{2H}_{1}+\frac{n^{4}\pi^{4}}{b^{4}}{\left(D_{22}\right)}_{p}}$$

(15c) $$\begin{array}{l} P_{\mathrm{mn}}=\frac{4}{a*b}\int_{0}^{b}\int_{0}^{a}P\left(x,y\right)\text{sin}\frac{m\pi x}{a}\text{sin}\frac{n\pi y}{b}\mathrm{dxdy}\\ =\frac{4}{a*b}\left[-\frac{m_{y}\gamma_{m}^{2}+m_{x}\gamma_{n}^{2}}{\gamma_{m}\gamma_{n}}\left(\text{cos}\;\gamma_{m}\;x_{1}-\text{cos}\;\gamma_{m}\;x_{2}\right)\left(\text{cos}\;\gamma_{n}\;y_{1}-\text{cos}\;\gamma_{n}\;y_{2}\right)\right] \end{array}$$

(15d) $$\gamma_{m}=\frac{m\pi }{a}\;;\;\gamma_{n}=\frac{n\pi }{b}$$

## 4. Finite Element Analysis

The finite element method is a widely used and powerful tool for analyzing complex structures. It is capable of dealing with the piezoelectrical materials. Many researchers have modelled the piezoelectric actuation using the finite element method. A commercially available finite element software ANSYS has the ability to analyze the piezoelectrical materials. In this study, the finite element software ANSYS is adopted to investigate the deflection of a simply supported plate induced by the surface bonded piezoelectric actuators. To perform the ANSYS finite element analysis for the piezoelectric actuator bonded structure, SOLID 45 elements and SOLID 5 were used in the composite plate and piezoelectric actuators, respectively.

**Figure 4.**
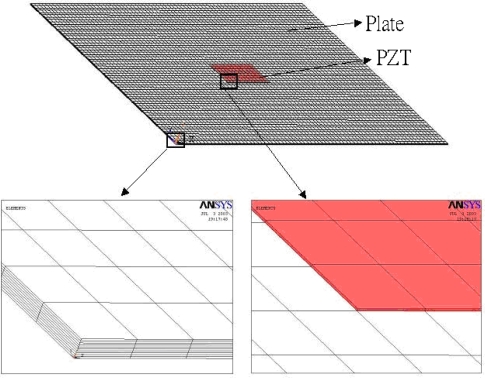
3-D finite element mesh.

A typical three dimensional finite element mesh is shown in Figure 4. A voltage between the upper and lower surfaces of the SOLID 5 elements is applied, which results in an electric field along the poling direction of the actuator. The deflections obtained from the finite element method are compared with the analytical solutions of Equation (15) to validate the present approach.

## 5. Numerical Validation and Examples

In the following numerical examples, the composite material is carbon/epoxy with stacking sequence [0/90/90/0].The composite material properties of carbon/epoxy are listed in Table 1. The dimensions of the composite laminate plate are length *a* = 0.38 m, width *b* = 0.3 m, thickness *t_p_* = 1.5876 mm. The piezoelectric actuator is assumed to be PZT G-1195 with the material properties [17] of Young’s modulus *E_pe_* = 63 GPa, Poisson’s ratio *v_pe_=* 0.3, density *ρ_pe_* = 7,600 kg/m^2^, piezoelectric constant *d_31_* = 1.9 × 10^−10^ V/m and thickness *t_pe_* = 0.15876 mm. The effects of the size and location of the actuators are presented through a parametric study to investigate the deflection and deformed shape of the composite plate activated by the surface bonded piezoelectric actuators.

### 5.1. Example 1: Three different sizes of actuators

**Figure 5.**
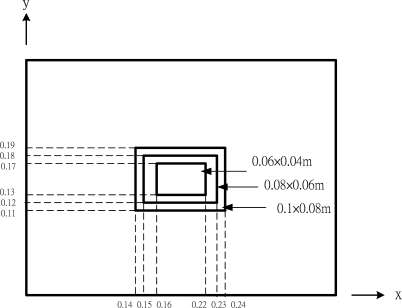
Three different sizes of PZT actuators.

Two piezoelectric actuators are surface bonded on the top and bottom surfaces of the composite plate. Three different sizes of piezoelectric actuators with the dimensions of 0.06 m × 0.04 m, 0.08 m × 0.06 m and 0.1 m × 0.08 m, respectively, bonded on the central area of the composite plate as shown in Figure 5 are considered in this example. The voltages of +1 V and −1 V are applied to the top and bottom actuators, respectively, resulting in a bending moment acting on the composite plate. The deflections of the composite plate induced by the actuators are calculated using both the analytical prediction of Equation (15) and finite element method.

**Figure 6.**
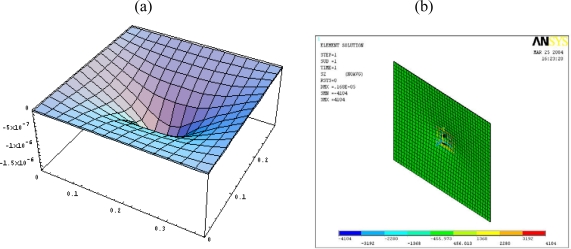
Deformation of the composite plate induced by the PZT actuator 0.06 m × 0.04 m obtained by (a) Equation (15) (b) finite element method (unit: m). (a) Deformed shape obtained by Equation (15). (b) Deformed shape obtained by ANSYS.

**Figure 7.**
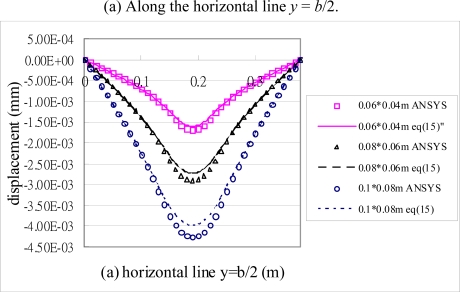

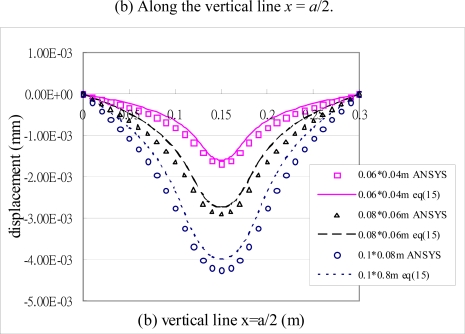
Displacements of the composite plate induced by PZT actuators with three different sizes.

Figure 6 shows the deformed shape of the composite plate predicted by Equation (15) and finite element method. The flexural displacements along the horizontal line *y = b*/2 and the vertical line *x = a*/2 of the composite plate are presented in Figure 7. The deflection is increasing as the size of actuators increases. The deflections of the composite plate predicted by Equation (15) and finite element method are agree well. The maximum deflections in the composite plate induced by the three different sizes of actuators are listed in Table 2. It shows that the difference between the present approach and finite element method is within 7 %. Similar difference was observed by Qing *et al.* [18] as comparing their semi-analytical solutions with ANSYS finite element results. The finite element methods can handle numerically a lot of problems in engineering field and has proved to be a powerful tool for the design and analysis of piezoelectric devices. Owing to the boundaries and geometric complexity of the adaptive structure, it is reasonable to validate the present model with finite element method.

### 5.2. Example 2: Three different locations of actuators

**Figure 8.**
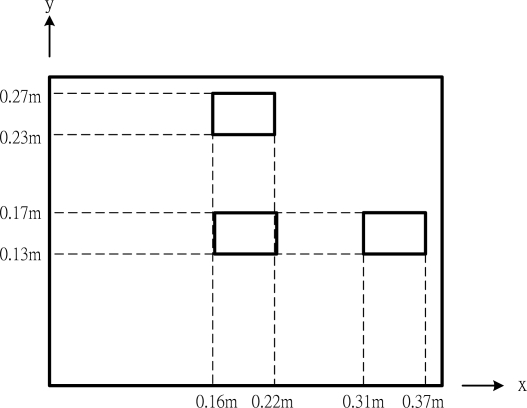
Three different locations of the actuator.

**Figure 9.**
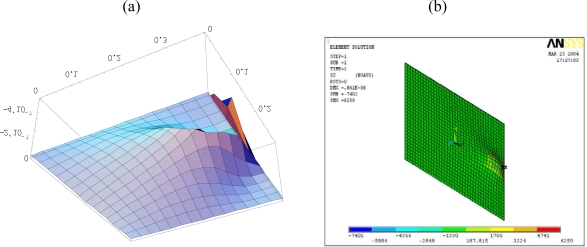
deformation of the composite plate induced by the PZT actuator bonded on the right region calculated by (a) Equation (15) (b) finite element method. (a) Deformed shape calculated by Equation (15). (b) Deformed shape calculated by ANSYS.

**Figure 10.**
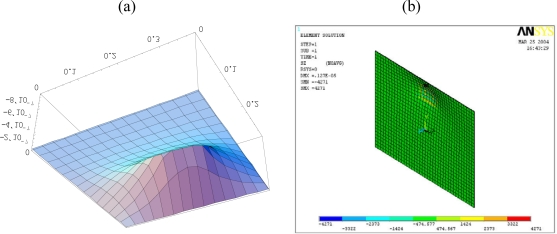
deformation of the composite plate induced by the PZT actuator bonded on the top region calculated by (a) Equation (15) (b) finite element method. (a) deformed shape calculated by Equation (15). (b) deformed shape calculated by ANSYS.

**Figure 11.**
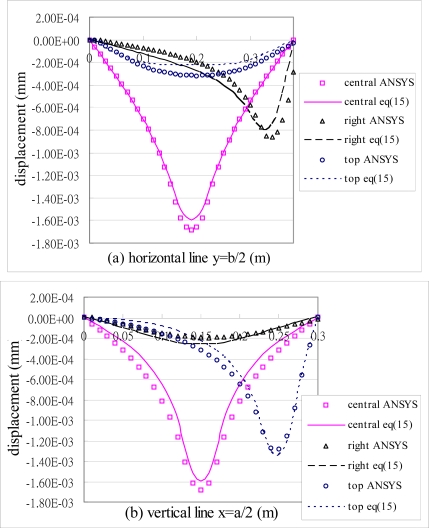
Flexural displacement of the composite plate obtained by ANSYS and Equation (15) along the horizontal line (*y = b*/2) and vertical line (*x = a*/2) for three different locations of actuator.

To study the capability of control the deflection shape of the plate, actuators are placed at various locations. In this example, the piezoelectric actuators are surface bonded at three different locations, central, right and top region of the plate, respectively, as shown in Figure 8. These three typical locations were arbitrarily chosen to demonstrate the influence of the actuator location on the deflection. The deformed shapes of the composite plate induced by the PZT actuators surface bonded at the central, right and top region of the plate are shown in Figures 6, 9 and 10, respectively. The difference of the deflected curves shown in Figures 6, 9 and 10 demonstrates that the shape of the plate can be controlled by placing the actuators at various locations. The flexural displacements of the plate along the horizontal line *y = b*/2 and vertical line *x = a*/2 are presented in Figure 11. The deflections of the plate obtained by the present approach of Equation (15) and finite element method are in close agreement. Table 3 lists the maximum deflections of the plate induced by the piezoelectric actuators surface bonding at three different locations. It shows that the difference between the present approach and finite element method is within 8%.

## 6. Conclusions

Piezoelectric materials are often used as strain actuators and shape control of smart structures, as they are compact and response quickly. In this investigation, two piezoelectric actuators are symmetrically surface bonded on a composite laminate plate. Electric voltages with the same amplitude and opposite sign are applied to the two symmetric piezoelectric actuators, resulting in the bending effect on the plate. Theoretical model of the bending moment is derived by using the theory of elasticity to represent the interaction of the actuator and the host plate. Following the classical plate theory, the deflection of a simply supported plate subjected to the bending moment can be obtained. The analytical solutions are validated with the finite element results. The effects of size and location of actuators on the responses of the plate are presented through the parametric study. Utilization of the laminate theory and plate theory, the deformed shape of the laminate plate can be predicted analytically. The methodology proposed in this paper is easy to employ, and provides an alternate way of solving this complicated problem analytically with accurate results.

## Figures and Tables

**Table 1.** Material properties of carbon/epoxy.

**Longitudinal modulus *E*_1_**	**Transverse modulus *E*_2_**	**Shear modulus *G*_12_**	**Shear modulus *G*_23_**	**Poisson’s ratio *v*_12_**	**Poisson’s ratio *v*_23_**
108 GPa	10.3 GPa	7.13 GPa	4.02 GPa	0.28	0.28

**Table 2.** maximum deflection of the composite plate induced by the PZT actuators with three different sizes.

**Method**	**ANSYS**	**Equation (15)**	**Error (%)**
**Size**			
PZT 0.06 m × 0.04 m	1.68 × 10^−3^ mm	1.59 × 10^−3^ mm	5.4
PZT 0.08 m × 0.06 m	2.90 × 10^−3^ mm	2.73 × 10^−3^ mm	6.3
PZT 0.1 m × 0.08 m	4.26 × 10^−3^ mm	3.98 × 10^−3^ mm	6.8

**Table 3.** maximum deflection of the composite plate induced by the PZT actuators at three different locations.

**Method**	**ANSYS**	**Equation (15)**	**Error (%)**
**Location**			
PZT at central region	1.68 × 10^−3^ mm	1.59 × 10^−3^ mm	5.4
PZT at right region	8.62 × 10^−4^ mm	8.00 × 10^−4^ mm	7.7
PZT at top region	1.27 × 10^−3^ mm	1.34 × 10^−3^ mm	4.9
